# Transgenic high expression of EphB4 in canine periodontal ligament stem cells modulates osteogenic differentiation and migration in cPDLSCs

**DOI:** 10.1186/s12938-026-01533-6

**Published:** 2026-02-09

**Authors:** Chang Shun Li, Xin Zhang, Hao Liu, Yan Feng, Lei Wang, Meng Zhou, Ni Na Xie, Shao Yue Zhu

**Affiliations:** 1 Department of Oral and Maxillofacial Surgery, Xuzhou Medical University’s Associated Dental Hospital, Xuzhou, Jiangsu 221000 People’s Republic of China; 2 Department of Pediatric Dentistry, Xuzhou Medical University’s Associated Dental Hospital, Xuzhou, Jiangsu 221000 People’s Republic of China; 3 Department of Orthodontics, Xuzhou Medical University’s Associated Dental Hospital, Xuzhou, Jiangsu 221000 People’s Republic of China; 4 Department of Periodontics, Xuzhou Medical University’s Associated Dental Hospital, Xuzhou, Jiangsu 221000 People’s Republic of China

## Abstract

**Background:**

Signaling molecules play a critical role in regulating stem cell-mediated bone regeneration. In this study, we investigated the effect of EphB4 gene transfection on the osteogenic differentiation and migratory capacity of canine periodontal ligament stem cells (cPDLSCs). Using a lentiviral vector, we established stable EphB4-overexpressing cPDLSCs and evaluated their functional properties in vitro and regenerative potential in vivo.

**Results:**

Our results demonstrated that EphB4 transfection significantly enhanced the osteogenic capability of cPDLSCs, as evidenced by elevated expression of osteogenic markers—osteocalcin, Runx2, and collagen type I—along with increased mineral deposition. Furthermore, EphB4 overexpression strongly promoted cell migration, indicating roles in enhancing cell homing and recruitment. Mechanistic analyses indicated that these effects were associated with greater EphB4 phosphorylation, suggesting activation of forward signaling pathways. In a canine alveolar bone defect model, transplantation of EphB4-modified cPDLSCs led to significantly augmented bone regeneration compared with control groups. Micro-computed tomography analysis revealed greater bone volume/total volume ratio, increased trabecular number and thickness, and reduced trabecular separation. Histological and immunohistochemical analyses confirmed greater expression of osteogenic proteins in the EphB4 group; no significant differences were observed between the two control groups (untreated cPDLSCs and empty vector-transduced cPDLSCs).

**Conclusions:**

These findings collectively indicate that EphB4 overexpression potentiates the osteogenic and migratory properties of cPDLSCs, thereby promoting alveolar bone repair in vivo. Our results highlight the therapeutic potential of EphB4 for periodontal and bone regeneration applications.

**Supplementary Information:**

The online version contains supplementary material available at 10.1186/s12938-026-01533-6.

## Introduction

The oral cavity offers a convenient and diverse reservoir of stem cells. Previous studies have demonstrated that the diversity of mesenchymal stem cells (MSCs) within this environment can be characterized based on their distinct tissue origins. Examples include stem cells from deciduous teeth, dental pulp (DPSCs), periodontal ligament (PDLSCs), apical papilla, gingival tissue, and the dental follicle [1]. DPSCs have demonstrated the ability to differentiate into multiple lineages. Bone grafting is widely recognized as an effective clinical strategy to improve the cosmetic and functional outcomes of skeletal deficiencies. Autologous bone grafting is favored clinically [2]. However, this approach presents several limitations, including limited donor tissue availability, potential resorption of grafted bone, and donor site morbidity [3]. Our previous study showed that ephrinB2 gene-modified canine PDLSCs (i.e., cPDLSCs) exhibit enhanced osteogenic differentiation. This feature arose from amalgamation of reverse signaling via ephrinB2 and forward signaling through EphB4. EphrinB2 alteration was also associated with increased proliferation and migratory activity in cPDLSCs [4]. In support of these findings, beagle-centered experiments revealed that ephrinB2 facilitates bone regeneration by enhancing osteogenesis in periodontal ligament stem cells, suggesting therapeutic applications in alveolar bone repair [5].

Bone tissue engineering, which integrates seed cells, scaffold materials, extracellular matrix, and growth factors, offers innovative therapeutic strategies for bone regeneration [6]. Among candidate cell types, MSCs are indispensable because of their ability to differentiate into bone-forming cells, as well as for their self-renewal capacity. cPDLSCs are distinguished from other MSCs by their superior osteogenic differentiation ability and proliferation rate [7–9], along with their broader availability and lower immunogenicity [10]. These characteristics make cPDLSCs attractive for use as seed cells in bone repair-targeting regenerative strategies [11].

EphB4, a member of the Eph receptor tyrosine kinase family, interacts specifically with its ligand ephrinB2 and modulates numerous biological mechanisms from embryogenesis through adulthood. The molecular interaction between EphB4 and ephrinB2 serves as an essential modulator of cellular behavior across a broad spectrum of developmental and physiological contexts. The precise regulation of cellular adhesion and repulsion mechanisms is central to these ligand–receptor interactions [12]. Generally, Eph-mediated forward signaling enhances cellular repulsion, whereas ephrin-mediated reverse signaling may induce either repulsion or adhesion, depending on the cellular environment and nature of the physical interaction. The ephrinB2-mediated reverse signaling cascade strongly inhibits osteoclast function through downregulation of key transcriptional regulators, particularly c-Fos and nuclear factor of activated T cells 1 (NFATC1), thereby suppressing osteoclast differentiation [13].

The ephrinB2/EphB4 signaling axis also plays a pivotal role in the regulation of mechanical forces involved in bone formation [14]. Disruption of this pathway has been shown to impair osteoblast activity through altered gene expression and mineralization processes. Osteoblast-derived ephrinB2 interacts with its cognate receptor EphB4, whereas PDLSCs express both ephrinB2 and EphB4, enabling crosstalk via paracrine or autocrine mechanisms. This interaction activates the receptor in a manner responsive to mechanical stimuli, as demonstrated in models involving Karli and lipopolysaccharide [15]. Notably, EphB4-Fc constructs have been shown to induce the expression of Runx2, a critical transcription factor, as well as alkaline phosphatase (ALP) in PDL fibrin matrices, implying a direct role in aiding PDLSC differentiation toward the osteogenic lineage [16]. However, the short half-life of recombinant EphB4-Fc proteins presents a challenge for the maintenance of stable biological effects.

The strategy of EphB4 overexpression in PDLSCs presents a promising alternative for enhancing osteogenic regeneration, addressing the limitations associated with exogenous EphB4-Fc application. In support of this concept, we generated EphB4-modified cPDLSCs (i.e., EphB4-cPDLSCs) and assessed the impact of this modification on their osteogenic potential under controlled in vitro conditions.

## Results

### Characterization of cPDLSCs

**Fig. 1:**
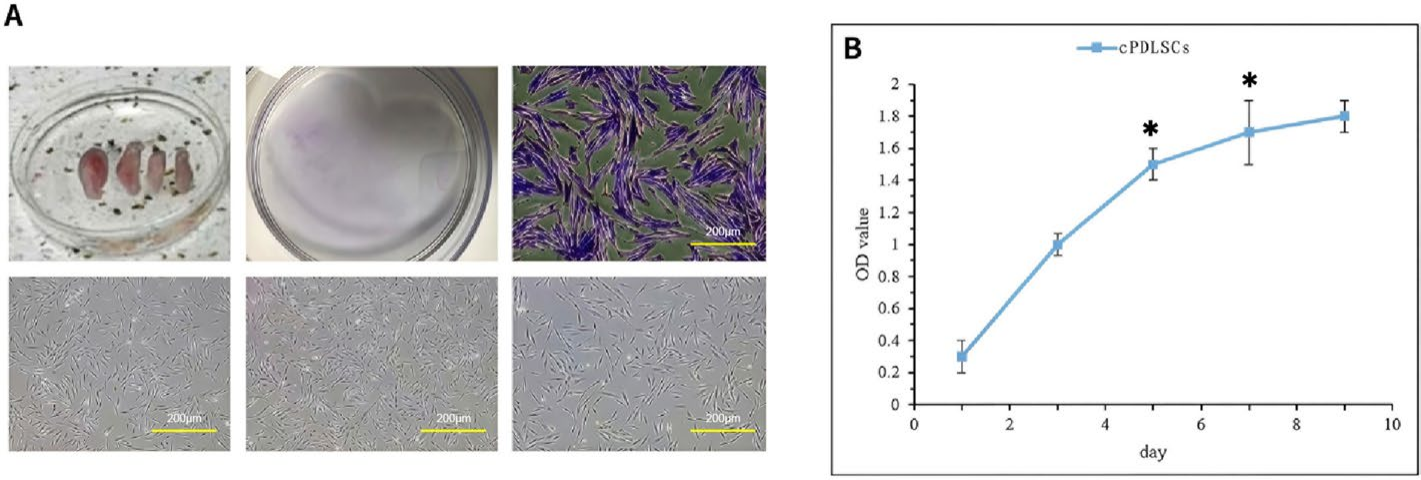
Isolation and characterization of canine periodontal ligament stem cells (cPDLSCs). **A** Morphological and clonogenic assessment of cPDLSCs. Left: primary cells isolated from periodontal ligament tissue exhibited typical fibroblast-like spindle morphology (original magnification: × 100). Right: crystal violet staining of colony-forming units after 10 days of culture (original magnification: × 40). **B** Proliferation kinetics of cPDLSCs measured by CCK-8 assay. Cells were seeded at a density of 2 × 10^3^ cells/well in 96-well plates, and absorbance at 450 nm was recorded daily for 7 days (*n* = 6 wells per time point). Values represent mean ± standard deviation (SD) from three independent experiments. Statistical significance was analyzed by one-way analysis of variance (ANOVA; *p < 0.05, **p < 0.01 compared with day 1)

Microscopic observation revealed that the isolated cells exhibited a conical and arborized morphology, with a strong tendency to adhere and proliferate along the culture surface. Colony-forming ability was documented via crystal violet marking (Fig. 1A), and the CCK-8 assay revealed an exponential proliferation curve, indicating robust cellular growth (Fig. 1B).

### Isolated cPDLSCs exhibit stem cell characteristics

**Fig. 2:**
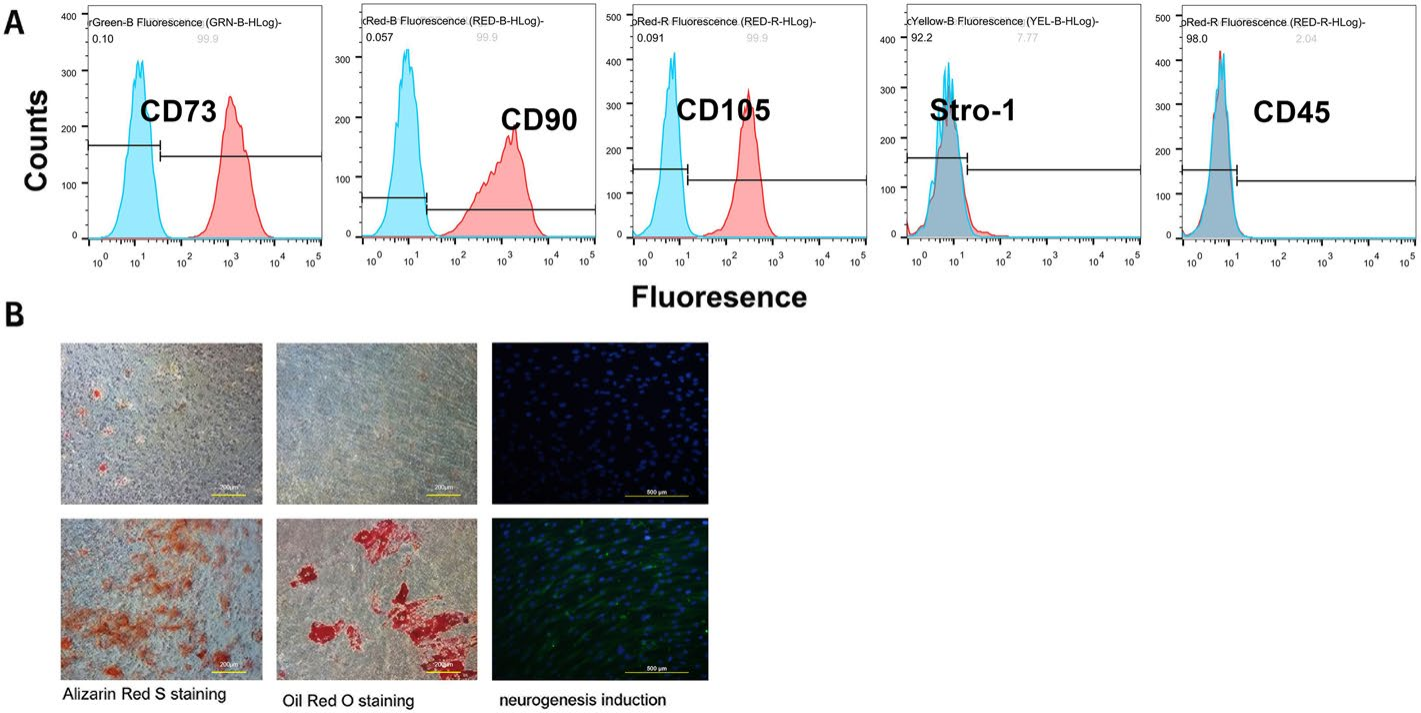
Characterization of canine periodontal ligament stem cell (cPDLSC) phenotypes and multilineage differentiation potential. **A** Flow cytometric analysis of surface marker expression in cPDLSCs at passage 3. Cells displayed positivity for mesenchymal stem cell markers CD90 (99.9%), CD105 (99.9%), and CD73 (99.9%); they showed negativity for the hematopoietic marker CD45 (2.04%). The early osteoprogenitor marker STRO-1 was detected at moderate levels in a subset of the population (7.77%). Representative histograms are shown with corresponding isotype controls in shaded curves. **B** Multilineage differentiation capacity of cPDLSCs. Osteogenic differentiation: Alizarin Red S staining revealed abundant calcium deposition after 21 days of induction (original magnification: × 100). Adipogenic differentiation: lipid vacuoles were visualized by Oil Red O staining after 14 days of adipogenic induction (original magnification: × 200). Neurogenic differentiation: immunofluorescence staining confirmed expression of the early neuronal marker βIII-tubulin (Tuj1, green) after 7 days of neurogenic induction. Nuclei were counterstained with 4′,6-diamidino-2-phenylindole (DAPI; blue) (original magnification: × 400)

Pivotal stem cell surface markers on cPDLSCs were typified by flow cytometry (Fig. 2A). The markers cluster of differentiation (CD)45, CD90, CD73, CD105, and STRO-1 were assessed to determine cPDLSC similarity to MSCs. A small proportion of cells expressed CD45 (2.04%), whereas high expression levels were observed for CD73 (99.9%), CD90 (99.9%), and CD105 (99.9%). STRO-1 expression was moderate (7.77%). Oil Red O-positive lipid vacuoles were presumed to verify adipogenic differentiation, whereas Alizarin Red S-marked nodules were deduced to constitute osteogenic differentiation (Fig. 2B). During neural induction, morphological alterations consistent with neuronal phenotype were observed alongside increased levels of βIII-tubulin, a neuron-specific marker.

### EphB4 gene-modified cPDLSCs exhibit GFP expression and elevated EphB4 mRNA and protein levels

**Fig. 3:**
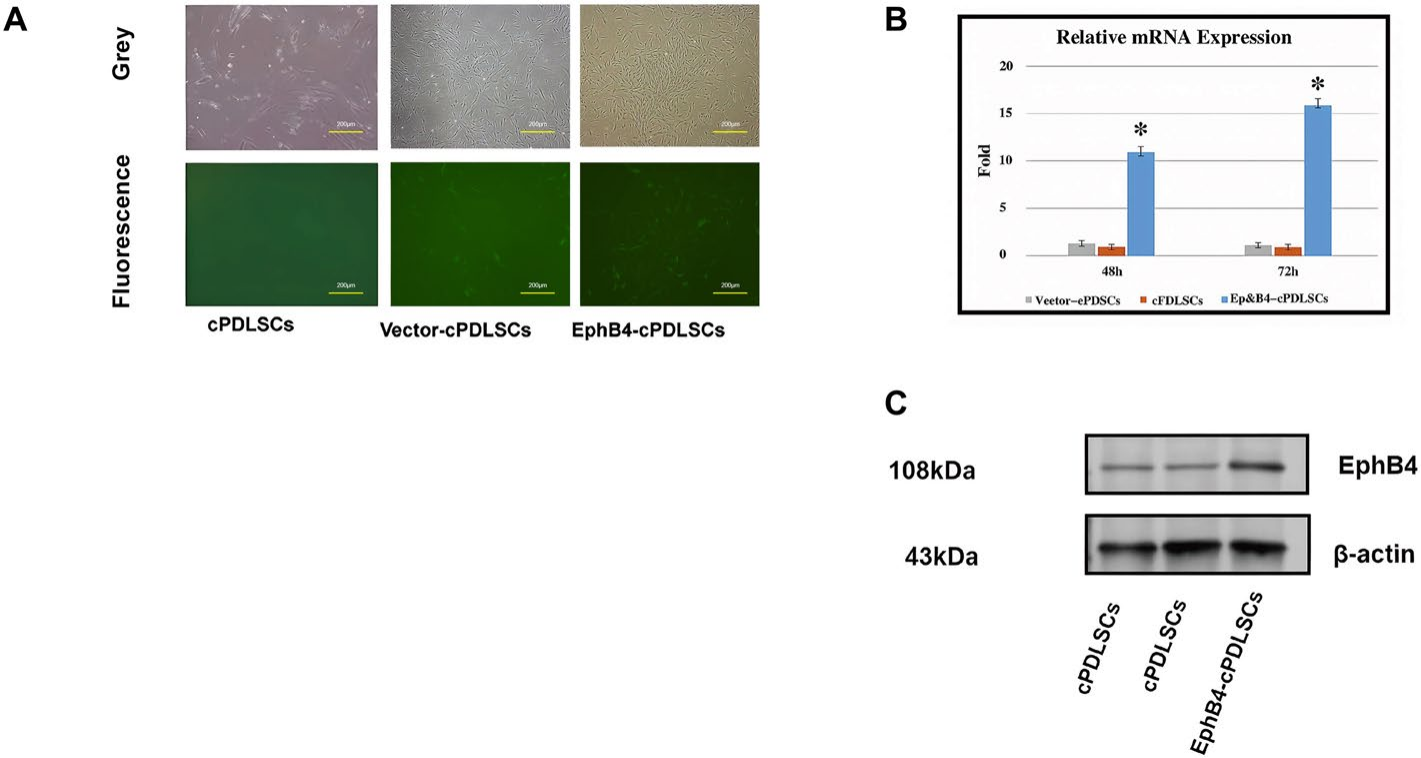
Validation of lentivirus-mediated EphB4 gene transfection and overexpression in cPDLSCs. **A** Fluorescence micrographs depicting robust GFP expression (green) in EphB4-transduced (EphB4-cPDLSCs) and empty vector-transduced (Vector-cPDLSCs) groups, indicating high transduction efficiency. Wild-type cPDLSCs (WT-cPDLSCs) showed no GFP signal. Scale bar: 100 μm. **B** Quantitative analysis of EphB4 expression at transcriptional and translational levels. Left: relative EphB4 mRNA expression normalized to GAPDH and quantified by RT-qPCR (*n* = 3, mean ± SD; **p < 0.01 vs. Vector-cPDLSCs). Right: relative EphB4 protein expression normalized to β-actin and quantified by western blot (*n* = 3, mean ± SD; *p < 0.05, **p < 0.01). **C** Representative western blot images demonstrating EphB4 protein expression in wild-type, empty vector-transduced, and EphB4-overexpressing cPDLSCs. β-Actin served as a loading control

GFP expression patterns in wild-type cPDLSCs, EphB4-cPDLSCs, and Vector-cPDLSCs were assessed by immunocytochemistry. As shown in Fig. 3A, wild-type cPDLSCs displayed no detectable fluorescence; Vector-cPDLSCs and EphB4-cPDLSCs both exhibited clear GFP expression under fluorescence microscopy. Quantitative analysis of EphB4 gene expression by RT-qPCR revealed a greater than 20-fold increase in mRNA levels in EphB4-cPDLSCs relative to wild-type and Vector-cPDLSCs (Fig. 3B). Western blot analysis confirmed significantly higher EphB4 protein levels in EphB4-cPDLSCs compared with the other two groups (Fig. 3C).

### EphB4 overexpression enhances cPDLSC migratory capacity

**Fig. 4:**
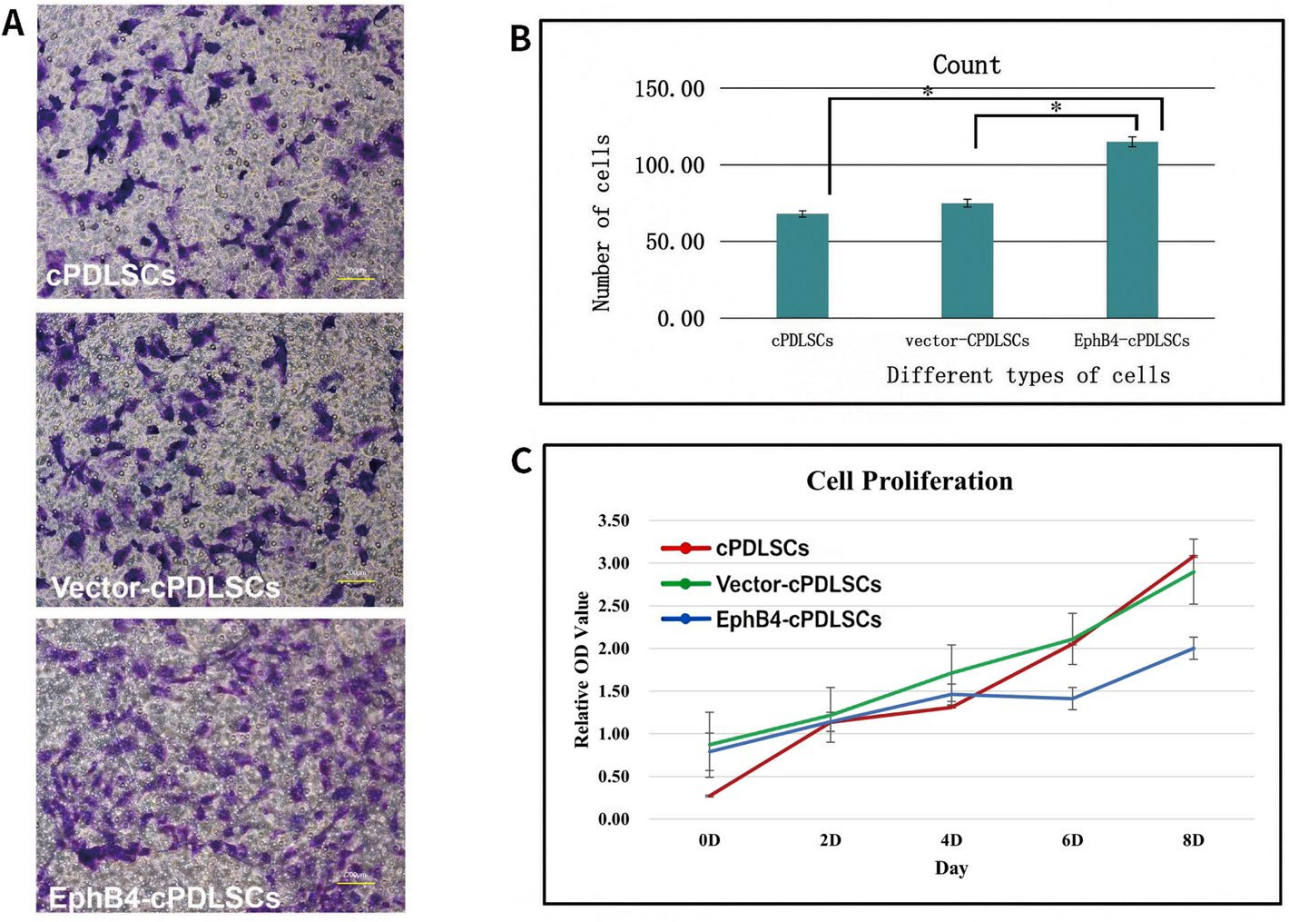
EphB4 overexpression enhances the migratory capacity of cPDLSCs without affecting proliferation. **A** Representative images of migrated cPDLSCs on the underside of Transwell membranes after 18 h of incubation (original magnification: × 100). From left to right: wild-type cPDLSCs, Vector-cPDLSCs, and EphB4-cPDLSCs. **B** Quantitative analysis of cell migration. Migrated cells were counted from five random fields per well (*n* = 3 independent experiments). Values represent mean ± SD; *p < 0.05 relative to both wild-type and Vector-cPDLSCs (one-way ANOVA with Tukey’s post hoc test). **C** Cell proliferation according to CCK-8 assay. Absorbance at 450 nm was measured at 24, 48, and 72 h after seeding (*n* = 6 wells per group). No significant differences in proliferation rates were observed among wild-type, Vector-cPDLSCs, and EphB4-cPDLSCs (mean ± SD; *p* > 0.05, one-way ANOVA)

EphB4-cPDLSCs demonstrated a substantial increase in migration capacity compared with both Vector-cPDLSCs and wild-type cPDLSCs, as indicated by statistically significant differences (*p* < 0.05; Fig. 4A, B). In contrast, the three groups did not show significant variation in propagation rates (Fig. 4C).

### EphB4 gene-modified cPDLSCs exhibit enhanced osteogenic potential

**Fig. 5:**
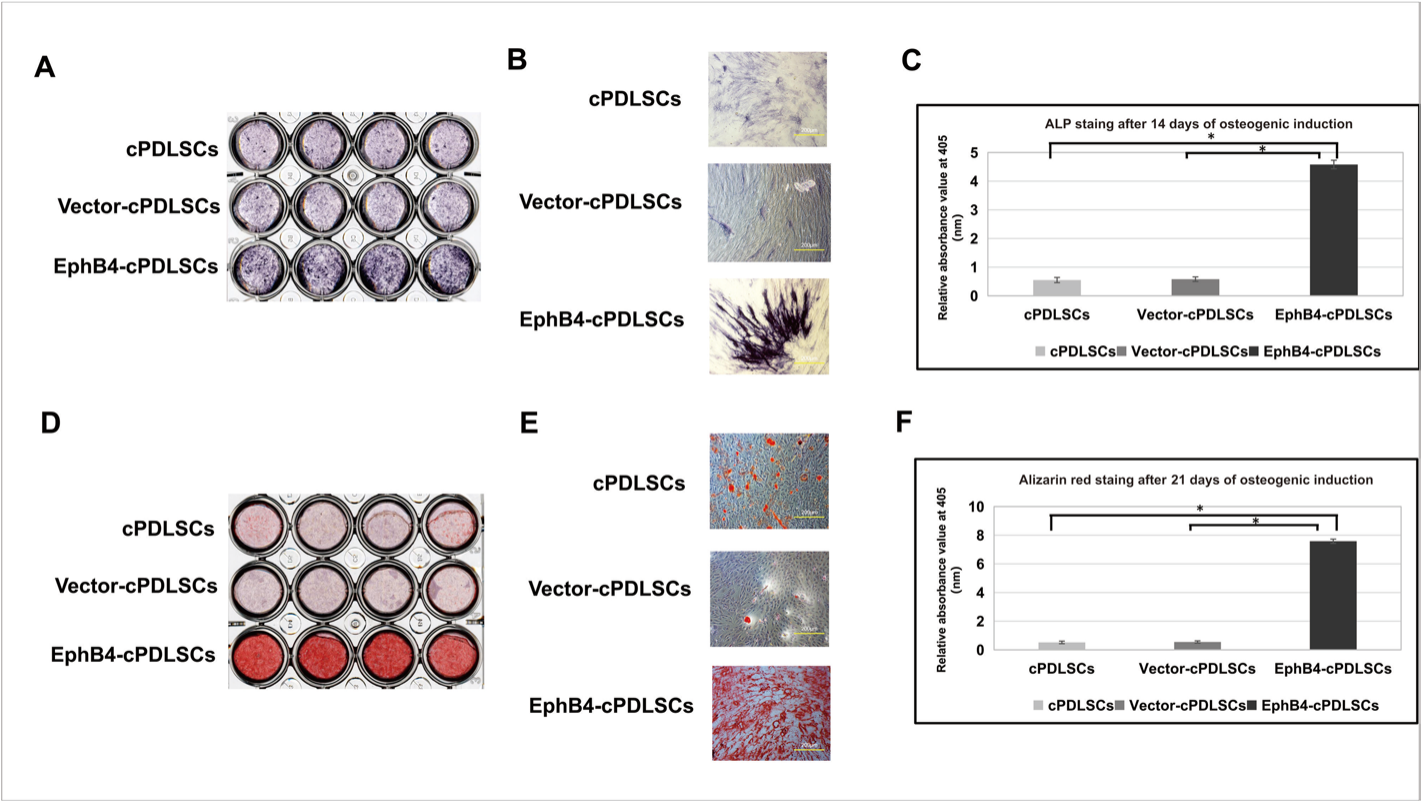
In vitro osteogenic differentiation potential of cPDLSCs under various conditions. **A** Alkaline phosphatase (ALP) staining of wild-type cPDLSCs (left), Vector-cPDLSCs (middle), and EphB4-cPDLSCs (right) after 14 days of osteogenic induction (original magnification: × 100). **B** Representative phase-contrast micrographs corresponding to the groups shown in (A) (original magnification: × 100). **C** Quantitative analysis of ALP activity measured by p-nitrophenyl phosphate hydrolysis and normalized to total protein content. Absorbance was measured at 405 nm. Values represent mean ± SD (*n* = 3; *p < 0.05, **p < 0.01 vs. Vector-cPDLSCs group; one-way ANOVA with Tukey’s post hoc test). **D** Alizarin Red S (ARS) staining of wild-type cPDLSCs (left), Vector-cPDLSCs (middle), and EphB4-cPDLSCs (right) after 14 days of osteogenic induction (original magnification: × 200). **E** Representative phase-contrast micrographs of mineralized nodules corresponding to the groups shown in (**D**) (original magnification: × 200). **F** Quantitative analysis of mineralization. Mineralization was quantified by eluting bound ARS with 10% cetylpyridinium chloride and measuring absorbance at 405 nm. Values represent mean ± SD (*n* = 3; *p < 0.05, **p < 0.01 vs. Vector-cPDLSCs group; one-way ANOVA with Tukey’s post hoc test)

ALP activity, evaluated on day 14 of osteogenic induction, was considerably higher in EphB4-cPDLSCs than in vector controls (Fig. 5A–D). By day 21, widespread mineralized deposits were more prominent in EphB4-cPDLSCs (Fig. 5E, F), indicating enhanced matrix mineralization.

### Overexpression of EphB4 elevates osteogenic marker expression

**Fig. 6:**
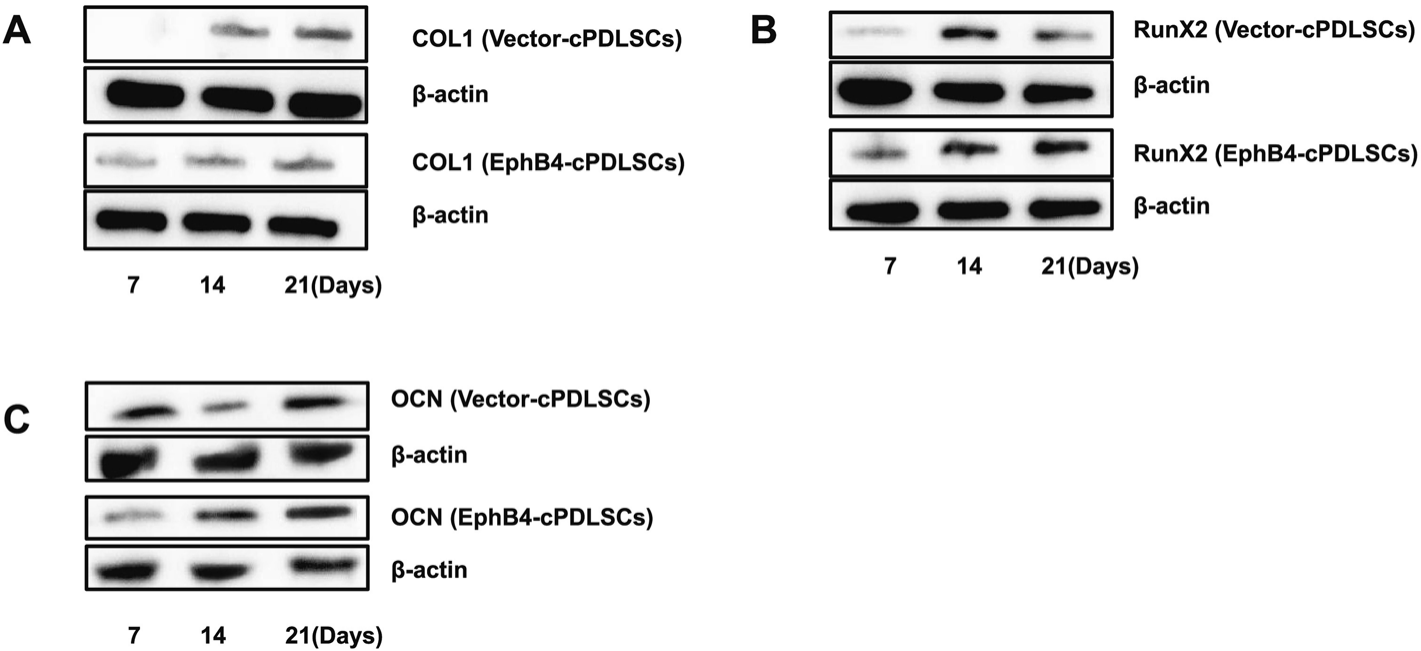
Western blot analysis of Runx2, COL1, and OCN expression during osteogenic differentiation in Vector- and EphB4-transfected cPDLSCs. **A** Representative western blot images of collagen type I (COL1) protein expression in Vector-cPDLSCs and EphB4-cPDLSCs at 7, 14, and 21 days of osteogenic induction. **B** Representative western blot images of Runx2 protein expression under the same conditions. **C** Representative western blot images of osteocalcin (OCN) protein expression under the same conditions. β-Actin served as a loading control

As illustrated in Fig. 6A–C, EphB4-cPDLSCs displayed substantially elevated expression levels of COL1, OCN, and Runx2 proteins compared with Vector-cPDLSCs after osteogenic induction.

### EphB4 phosphorylation showed distinct temporal patterns between groups

**Fig. 7:**
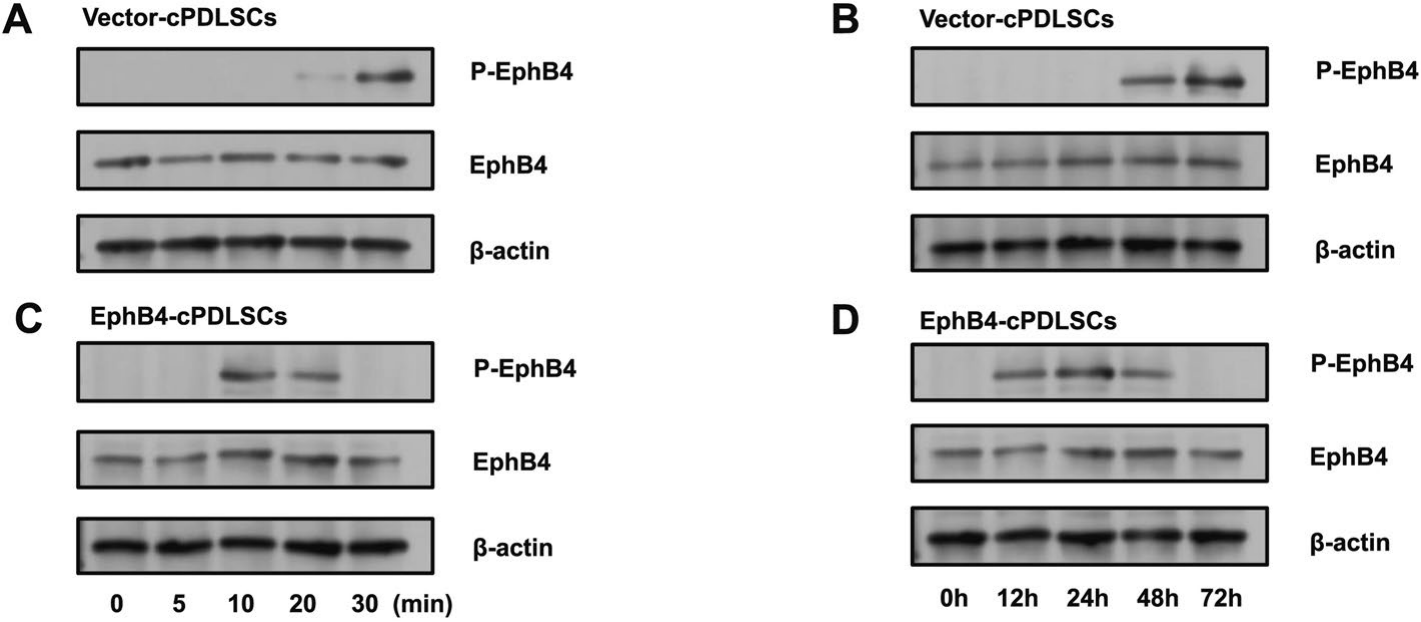
Temporal dynamics of EphB4 phosphorylation in EphB4-cPDLSCs versus Vector-transfected controls. **A** In Vector-cPDLSCs, EphB4 phosphorylation was not detected until 30 min after the onset of osteogenic induction. **B** In Vector-cPDLSCs, weak EphB4 phosphorylation appeared at 48 h and subsided within 24 h. **C** Time-course analysis of EphB4 phosphorylation in Vector-cPDLSCs within 72 h of osteogenic induction. **D** Time-course analysis of sustained EphB4 phosphorylation in EphB4-cPDLSCs within 72 h of osteogenic induction. Total EphB4 and β-actin served as internal controls. All blots represent findings from three independent experiments

In Vector-cPDLSCs, EphB4 phosphorylation was not detected until 30 min after the onset of osteogenic induction (Fig. 7A). In addition, EphB4 phosphorylation appeared at 48 h in Vector-cPDLSCs, then subsided within 24 h (Fig. 7B). In contrast, EphB4-cPDLSCs exhibited detectable phosphorylation as early as 20 min, with higher initial phosphorylation levels (Fig. 7C). In EphB4-cPDLSCs, phosphorylation was observed at 12 h and persisted for 24 h (Fig. 7D).

### Osteoblastic differentiation compels ephrinB2/EphB4 signaling in vitro

**Fig. 8:**
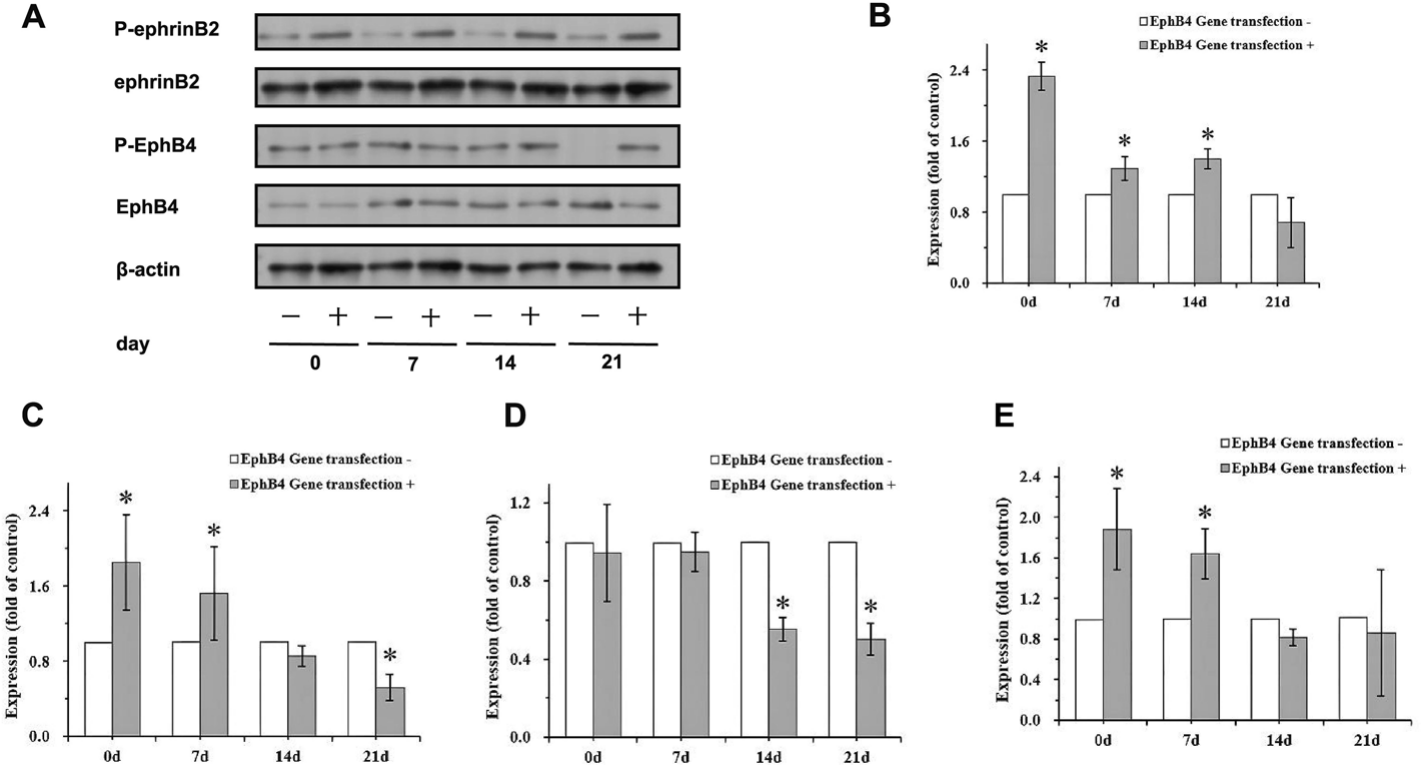
Phosphorylation levels of EphB4 (p-EphB4), ephrinB2, and phosphorylated ephrinB2 (p-ephrinB2) in EphB4-cPDLSCs and Vector-cPDLSCs under osteogenic induction. **A** Representative western blot images showing protein expression of total and phosphorylated forms of ephrinB2 and EphB4 at 0, 7, 14, and 21 days after osteogenic induction. β-Actin served as a loading control. **B** Densitometric quantification of ephrinB2 protein expression normalized to β-actin. Values represent mean ± SD from three independent experiments. **C** Densitometric quantification of EphB4 protein expression normalized to β-actin. **D** Densitometric quantification of p-ephrinB2 protein expression normalized to total ephrinB2. **E** Densitometric quantification of p-EphB4 protein expression normalized to total EphB4. *p < 0.05, **p < 0.01, ***p < 0.001 indicate statistically significant differences between EphB4-cPDLSCs and Vector-cPDLSCs at each time point (two-way ANOVA with Bonferroni post hoc test)

Under osteogenic induction, expression levels of ephrinB2, phosphorylated ephrinB2 (p-ephrinB2), EphB4, and phosphorylated EphB4 (p-EphB4) were significantly upregulated in cPDLSCs. Western blot analyses comparing EphB4-cPDLSCs and Vector-cPDLSCs at days 0, 7, 14, and 21 post-induction revealed progressive increases in expression levels (Fig. 8A; densitometry conveyed in Fig. 8B–E).

### Histological analysis

**Fig. 9:**
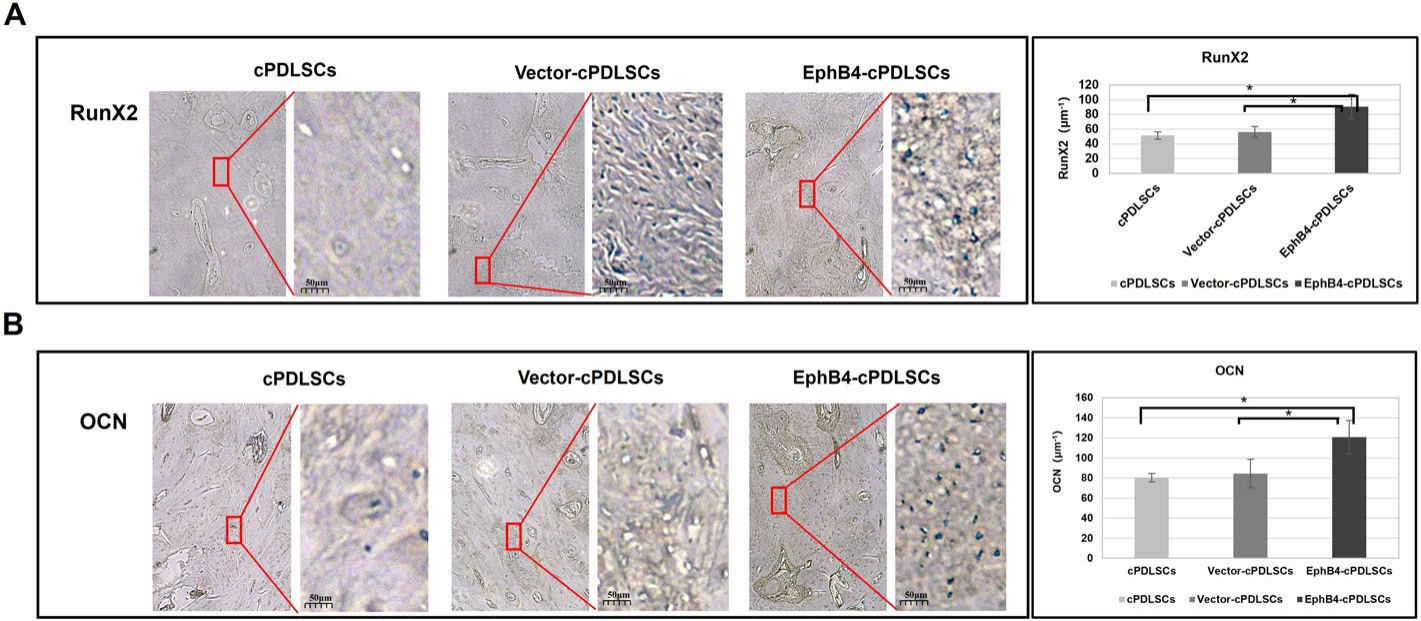
Immunohistochemical analysis of osteogenic marker expression in regenerated alveolar bone. Representative immunohistochemical staining for **A** Runx2 and **B** osteocalcin (OCN) in alveolar bone defects treated with EphB4-cPDLSCs, Vector-cPDLSCs, or untreated cPDLSCs (original magnification: × 200). Insets show higher magnification views of areas indicated by red squares, highlighting protein-positive cells. **C** Quantitative analysis of Runx2-positive cells and (D) OCN-positive cells in the region of interest. The EphB4-cPDLSCs group exhibited significantly increased positive staining for both markers relative to both control groups (*p < 0.05, **p < 0.01). No statistically significant difference was observed between the Vector-cPDLSCs and untreated cPDLSCs groups. Values represent mean ± SD; *n* = 6 per group; one-way ANOVA with Tukey’s post hoc test

Histological evaluation revealed significant increases in Runx2- and OCN-positive cell numbers within the region of interest in the EphB4-cPDLSCs group relative to the untreated cPDLSCs and Vector-cPDLSCs control groups. No statistically significant difference was observed between the two control groups. Representative images and quantitative data are presented in Fig. 9.

### Micro-computed tomography

**Fig. 10:**
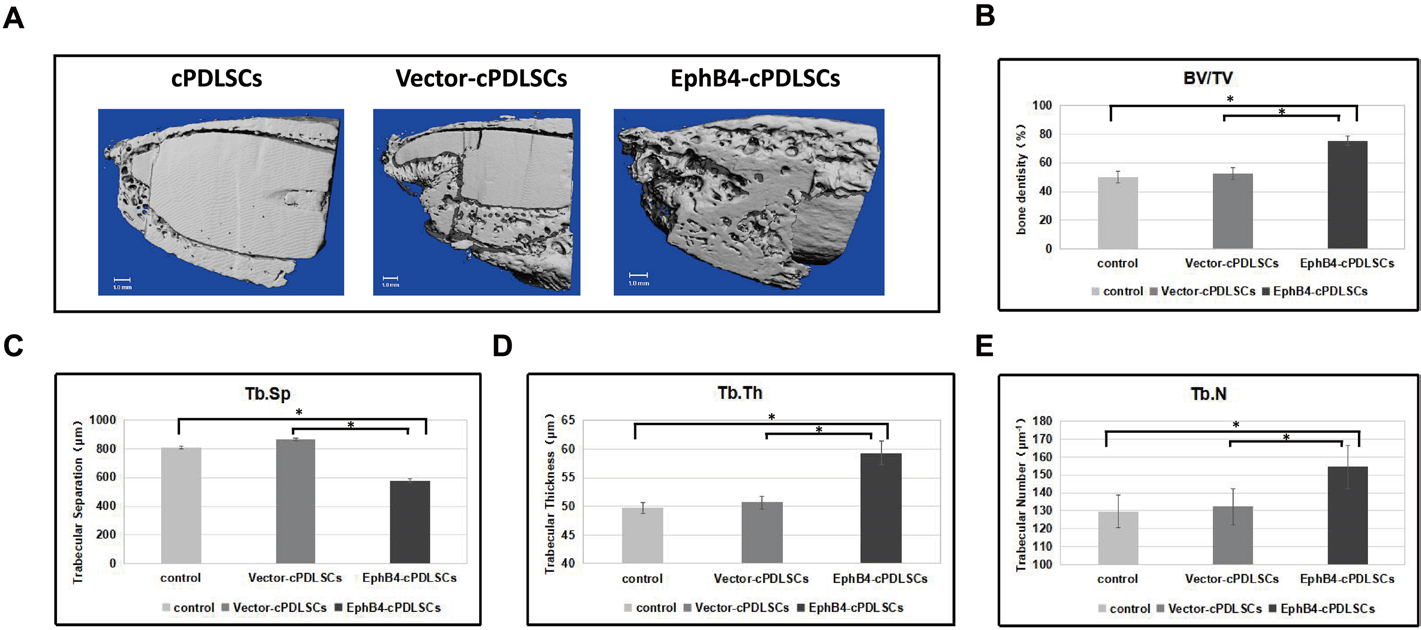
Micro-CT. **A** Representative 3D reconstructed micro-CT images of the distal femur from each group. Yellow dashed box indicates the region of interest for quantitative analysis. Scale bar: 1 mm. **B**–**E** Quantitative morphometric analysis of trabecular bone: **B** bone volume/total volume (BV/TV, %), **C** trabecular separation (Tb.Sp, μm), **D** trabecular thickness (Tb.Th, μm), and **E** trabecular number (Tb.N, mm^−1^). Values represent mean ± SD (*n* = X). Statistical significance was determined by one-way ANOVA with Tukey’s post hoc test. *p < 0.05, **p < 0.01, ***p < 0.001 vs. [Control Group]; #*p* < 0.05, ##*p* < 0.01 vs. [Group Name]

Quantitative micro-computed tomography (CT) analysis revealed significantly enhanced bone regeneration in the EphB4-cPDLSCs group compared with both control groups. The experimental group demonstrated a significantly bone volume/total volume (BV/TV) ratio, along with improved trabecular microarchitecture, as evidenced by significant increases in trabecular number (Tb.N) and trabecular thickness (Tb.Th), and a concurrent decrease in trabecular separation (Tb.Sp). Notably, the Tb.Sp value was lowest in the EphB4-cPDLSCs group, indicating a denser and more connected trabecular network. No statistically significant differences in these parameters were observed between the cPDLSCs sheet and Vector-cPDLSCs sheet groups. Representative three-dimensional (3D) reconstructions and quantitative data are presented in Fig. 10A–E.

## Discussion

Regenerative therapies utilizing stem cells in periodontics rely on two principal modes of action. One involves delineation of transplanted stem cells into pivotal cell types that promote structural restoration of periodontal tissues. The other leverages their use as transporters for targeted delivery of therapeutic genes. This dual functionality makes stem cell therapy a compelling approach for enhancing periodontal repair. Gene therapy in this context involves either direct in situ administration of viral or non-viral vectors to the lesion site, or ex vivo modification followed by gene-loaded carrier cell transplantation [17–19]. Direct in situ gene transfer requires sufficient transduction of healthy cells at the site of injury, followed by appropriate endogenous transgene expression [20]. However, this approach is often impractical in injured periodontal tissue because of the scarcity of viable stem cells within the inflammatory and pathogenic microenvironment [21]. The use of transplanted autologous stem cells as gene delivery vehicles may overcome these challenges by combining the regenerative potential of stem cells with the therapeutic benefits of gene transfer. Progenitor cells and MSCs are particularly advantageous because of their dual capacity to deliver bioactive genes and regenerate periodontal structures. Stem cells derived from dental tissues exhibit greater plasticity than MSCs from non-dental sources, making them more suitable for differentiation into dental and oral cell lineages [22, 23]. For example, collagen produced by non-oral MSCs may diverge in numerous ways.

Adult bone is a dynamic tissue, continuously remodeled through coordinated bone formation and resorption, which are essential for maintaining its morphology and function [24]. Zhao and colleagues identified a bidirectional signaling mechanism involving the ephrinB2 ligand and its receptor, EphB4 [25, 26]. This pathway balances bone resorption and formation. Specifically, reverse signaling via ephrinB2 inhibits osteoclast activity by downregulating the transcription factors c-Fos and NFATC1, leading to reduced bone resorption. Concurrently, EphB4 forward signaling lowers RhoA movement, authorizing osteoblast differentiation and inciting bone matrix deposition. These effects have been confirmed through a combination of cell-based assays and animal studies.

Cell-based studies have shown that ephrinB2-Fc stimulates mouse bone marrow stromal cells (ST2 cells). Notably, ablation of the EphB4 gene led to a significant increase in the osteogenic differentiation of ST2 cells, affecting their bone-forming capacity [27]. Animal studies further demonstrated that mice with defective ephrinB2 expression exhibited reduced bone mineralization, decreased bone rigidity, and altered osteoblast characteristics relative to wild-type controls [4].

PuraMatrix, a synthetic self-assembling peptide hydrogel, reportedly provides a supportive microenvironment enabling survival and differentiation of MSCs and progenitor cells [28]. In earlier work, we found that cPDLSCs encapsulated in PuraMatrix gradually extended their processes and displayed increased cellular density over time, indicative of sustained viability and proliferation within the matrix. To determine whether PuraMatrix alone could influence bone regeneration, we included a control group in which the hydrogel was implanted without cells. Our results indicated that PuraMatrix by itself did not significantly affect bone regeneration [29]. Furthermore, a comparative analysis between EphB4 transgenic mice and wild-type controls revealed substantial differences in bone regeneration capacity. The transgenic mice exhibited enhanced new bone formation at fracture sites, along with a significant increase in the formation of clonal mesenchymal stromal progenitor cells [30]. Our previous study also demonstrated that ephrinB2 transfection of PDLSCs in vitro led to increased ALP activity, enhanced formation of calcified nodules, and upregulation of genes associated with osteoblast function. A key advantage of targeting the EphB4–ephrinB2 signaling pathway over canonical osteogenic factors (e.g., bone morphogenetic proteins) lies in its dual functionality. Although bone morphogenetic proteins are potent osteoinductors, they have been associated with complications, including ectopic bone formation and insufficient accompanying vascularization. In contrast, our work and others ([31, 32], including our previous studies) demonstrate that EphB4 promotes osteogenesis while playing a critical role in the formation of a stable and mature vascular network. This coordinated enhancement of bone formation and angiogenesis is essential for successful bone regeneration and metabolic balance.

## Conclusion

The present study demonstrated that cPDLSCs engineered to express EphB4 exhibit enhanced osteogenic differentiation capacity, and EphB4-mediated forward signaling occupies a central regulatory role in these effects. In addition, increased EphB4 expression significantly increased the migratory capacity of cPDLSCs, although it had no impact on proliferative ability.

## Methods

### Cell culture, isolation, and identification

cPDLSCs were isolated from four healthy 6-month-old beagle dogs (JC0853, JC0857, JC0889, JC0899; body weights of 6.6–8.0 kg), obeying practices stipulated by the Institutional Animal Care and Use Committee of Xuzhou Medical University (Consent Numeral 20,191,108, Xuzhou, China). Anesthesia was instituted utilizing intravenous propofol (6 mg/kg), pursued by 2% isoflurane maintenance. Oxygen was initially administered at 3 L/min, then reduced to 0.4 L/min. Bilateral mandibular third premolars were split via furcation and extracted. After 6 tooth extractions, the extraction site was thoroughly irrigated with sterile saline, packed with Bona medical collagen (Bedi Bioengineering Co., Ltd., Wuxi, China), and sutured using 4–0 polyglycolic acid absorbable sutures (Boda Medical Supplies Co., Ltd., Shandong, China). Postoperative antimicrobial prophylaxis was administered subcutaneously using cefotiam sodium (10 mg/kg; Quanyu Biotechnology Animal Pharmaceutical Co., Ltd., Shanghai, China). Anesthetic agents were withdrawn after the procedure; each animal was monitored for 30 min to ensure stable cardiopulmonary function and full recovery of consciousness. Once fully alert and exhibiting normal motor responses, the animals were observed for 1 h and then returned to their cages. During the following 24 h, feeding was provided; local wound care with 0.5% iodophor was performed every 6 h. The extricated maxillary and mandibular anterior teeth were cleaned in phosphate-buffered saline supplemented with streptomycin and penicillin to remove surface contaminants. Tissue digestion and cell isolation were performed under sterile conditions using a mixture of collagenase I (3 mg/mL) and neutral protease (4 mg/mL). The resulting solitary cells were propagated under accepted conditions (37 °C, 5% CO_2_) and discerned by means of a reversed microscope. Flow cytometry was conducted to confirm the cell phenotype using markers such as STRO-1, CD45, CD73, CD90, and CD105.

### Transfection and identification of EphB4 gene-transfected cPDLSCs

Stable EphB4-overexpressing canine periodontal ligament stem cells (cPDLSCs) were established by lentiviral transduction. The canine EphB4 coding sequence with a C-terminal 3 × FLAG tag was cloned into the pLV4ltr-PGK-ZsGreen(2A)Pur0-CMV-C3xFLAG-EphB4 transfer plasmid (KRS151, Corues Biotechnology, Nanjing, Jiangsu, China). Lentiviral particles were produced by co-transfecting HEK-293T cells (ATCC^®^ CRL-3216™) with the transfer plasmid and the packaging plasmids psPAX2 (Addgene #12260) and pMD2.G (Addgene #12259) using polyethylenimine. Viral supernatants were collected at 48–72 h, concentrated by ultracentrifugation, and stored at − 80 °C.

For transduction, cPDLSCs were infected at a multiplicity of infection of 20 in the presence of 8 μg/mL Polybrene (Sigma-Aldrich, H9268). After 48 h, successful transductants were selected using 2 μg/mL puromycin (Vicmed) for 7 days. Confirmation of successful transduction and selection was achieved through a multi-step approach: initial assessment of ZsGreen fluorescence to determine transduction efficiency, followed by western blot analysis to detect FLAG-tagged EphB4 protein, and RT-qPCR to quantify EphB4 mRNA levels.

### RT-PCR

For quantification of total EphB4 mRNA expression, total RNA was extracted from the three experimental groups: non-transduced cPDLSCs, empty vector-transduced cPDLSCs, and EphB4-transduced cPDLSCs. RT-qPCR amplification was performed using primers targeting the EphB4 sequence; the primer sequences are provided in Table 1. Gene expression was normalized to *GAPDH*, and the 2^−ΔΔCt^ method was used for quantification.

**Table 1:** Primers used in this study

Canine gene	Primers
EphB4	For: AGGAGCACCACAGCCAGACC Rev: GCAATGACAATGACCACCAGGAC
GAPDH	For: GGCATGGACTGTGGTCATGAG Rev: TGCACCACCAACTGCTTAGC

### Cell proliferation assay

To judge cell propagation, a cell counting kit (CCK-8) assay (Genomeditech, Shanghai, China) was used. Unmodified cPDLSCs, EphB4-overexpressing cPDLSCs, and vector control cPDLSCs were sown in 96-well plates at a density of 1 × 10^4^/well and incubated overnight under accepted conditions (37 °C, 5% CO_2_). At designated time points (days 0, 2, 4, 6, and 8), 10 μL of CCK-8 reagent was added to each well. Plates were returned to the incubator for 4 h and then subjected to absorbance determination using a microplate reader at 450 nm to clarify cell viability.

### Cell migration assay

Migratory behavior was surveyed using a Transwell migration system (Boyden chambers; BD LabWare). Inserts were positioned in 24-well plates. In the upper chambers, 1 × 10^4^ cPDLSCs, EphB4-cPDLSCs, or Vector-cPDLSCs were seeded in serum-free α-minimum essential medium (α-MEM). Following 12 h of serum deprivation, α-MEM supplemented with 15% fetal bovine serum was loaded into lower compartments to establish a chemoattractant gradient. Cells that journeyed through the membrane and clung to its underside were fixed, stained, and counted at 6-, 8-, 10-, and 12-h intervals to appraise migration capacity.

### Evaluation of osteogenic potential among cPDLSCs, EphB4-cPDLSCs, and Vector-cPDLSCs in vitro

To evaluate osteogenic potential, both unmodified cPDLSCs and EphB4-overexpressing cPDLSCs were cultured in osteogenic induction medium for 7, 14, or 21 days. Further details are provided later in the article. Protein expression pertinent to bone formation and vascular development was inspected via western blot analysis. Mineral deposition and ALP movement were evaluated through Alizarin Red S staining and ALP-specific assays. For ALP analysis on day 14, cells were handled with a BCIP/NBT ALP Color Development Kit (Beyotime Institute of Biotechnology), and enzyme movement was explored using a colorimetric assay kit (Jiancheng Bioengineering Institute). Cells were fixed on day 21 with 4% paraformaldehyde and then marked with 0.2% Alizarin Red S to document mineralized extracellular matrix. To quantify calcium deposition in EphB4-cPDLSCs and Vector-cPDLSCs, a 10% (w/v) sodium dodecyl sulfate (SDS) solution was applied to the cultures, followed by 37 °C incubation overnight. The extent of mineralization was documented via microscopy.

### Western blot analysis

In 6-well platters, vector-transfected and EphB4-overexpressing cPDLSCs were sown at a density of 1 × 10^4^/well. Cultures at 70% confluence were exposed to either EphB4-Fc protein (R&D Systems) at 2 µg/mL or osteogenic induction medium. Time-course experiments were conducted for various durations: EphB4-Fc stimulation for 0, 5, 10, 20, 30, or 60 min, and osteogenic induction for 0, 12, 24, 48, or 72 h. Protein extraction was performed using M-PER lysis buffer (Thermo Fisher Scientific) in conjunction with a protease inhibitor cocktail. Protein quantities were judged using a bicinchoninic acid protein assay kit (Thermo Fisher Scientific). Proteins were disconnected by SDS–polyacrylamide gel electrophoresis (7.5% or 12% gels) and then relocated to polyvinylidene fluoride membranes (EMD Millipore). Membrane blocking (1 h at room temperature) used 5% bovine serum albumin mixed with Tris-buffered saline containing 0.05% Tween-20 (TBS-T). Primary antibodies targeting ephrinB2 (ab131536), phospho-ephrinB2 (ab119323), EphB4 (sc-5536), phospho-EphB4 (ab12720), collagen I (COL1; ab6308), Runx2 (ab76956), and osteocalcin (OCN; 33-5400) were obtained from Abcam and Thermo Fisher Scientific. Incubation overnight at 4 °C was implemented for antibody application. After TBS-T washes (three total, 5 min each), membranes were subjected to 1 h of warming with Cell Signaling Technology-derived horseradish peroxidase-conjugated secondary antibodies against rabbit IgG (7074) or mouse IgG (7076). Additional TBS-T washes (three total) were performed prior to signal detection by means of enhanced chemiluminescence (ECL) substrate, followed by image acquisition using a chemiluminescence imaging system.

### Fabrication of EphB4-cPDLSCs 3D cell sheet

EphB4-cPDLSCs were cultured in temperature-responsive dishes (UpCell® CellSeed, Tokyo, Japan). After complete cell attachment, the culture medium was replaced with film-forming induction solution (10% fetal bovine serum, 50 mg/L vitamin C, α-MEM). Upon induction of sheet formation, the medium was replaced with osteogenic induction medium for continuous culture for 7 days. ALP activity and calcium nodule formation were assessed in each group at 14 and 21 days. Osteogenic and angiogenic capacities were evaluated, and the optimal group was selected to construct the 3D cell sheet. A 35-mm temperature-responsive dish (UpCell^®^ CellSeed) and a support membrane were used to construct the optimal mixed cell set as a 3D sheet with three cell layers. Three-dimensional cell membranes prepared from Vector-cPDLSCs served as controls. The procedure was as follows: when cells reached 90% confluence, dishes were brought to room temperature (20℃) and the culture medium was removed. After 45 min, a support membrane was placed over the cell surface to allow adhesion between the cells and the membrane. Using sterile forceps, the membrane with the attached cell layer was transferred to another dish. The dish was then placed in an incubator at 37 °C for 15 min to allow membrane separation from the cell layer. This procedure was repeated to obtain the final 3D cell sheet.

### Surgical procedures for animal experiments

All surgical procedures were performed on four adult beagles in accordance with the approved protocols of Xuzhou Medical University (Consent Number 20191108, Xuzhou, China). After a 3-month post-extraction healing period, bilateral critical-sized alveolar bone defects were created in the beagle mandible using standard surgical protocols. Through a mid-crestal incision between the second and fourth premolars (PM2–PM4) with full-thickness flap elevation, four-wall intrabony defects (4 × 2 × 5 mm) were prepared at the mesial aspect of PM4 and the distal aspect of PM2, maintaining a distance of 1–2 mm from adjacent teeth. This defect dimension is well established as a critical-sized model in canines and exhibits no spontaneous healing capacity. In total, four defects were created per animal.

The defects in each dog were randomly assigned to three treatment groups and implanted with the corresponding cell sheet constructs: (1) cPDLSCs cell sheet group, (2) Vector-cPDLSCs cell sheet group, and (3) EphB4-cPDLSCs sheet group. All defects were covered with a Bio-Gide collagen membrane before tension-free wound closure. Postoperative care included antibiotic administration for 3 days. Enhanced osteogenesis, a key aspect of the regenerative outcome, was characterized by immunohistochemical staining for Runx2 (Proteintech, 20700-1-AP) and OCN (Proteintech, 23418-1-AP).

### Micro-CT and histological analysis

At 8 weeks postoperatively, calvarial samples were harvested and fixed. Bone regeneration within the defects was evaluated using a high-resolution micro-CT scanner. Three-dimensional morphological parameters, including BV/TV, Tb.N, and Tb.Th, were analyzed using built-in software.

After micro-CT scanning, samples were decalcified, embedded in paraffin, and sectioned. For immunohistochemical analysis, sections were incubated with primary antibodies against Runx2 (Proteintech, 20700-1-AP) and OCN (Proteintech, 23418-1-AP) to assess osteogenic activity. Each antibody’s specificity for canine antigens was confirmed prior to the experiment.

### Statistical analysis

Outcomes are conveyed in mean ± standard deviation format. Statistical divergences were appraised through one-way analysis of variance, pursued by Bonferroni post hoc testing for pairwise contrasts. The statistical significance threshold was regarded as *p* < 0.05.

## Supplementary Information

Supplementary Material 1.

Supplementary Material 2.

Supplementary Material 3.

## Data Availability

Data supporting the present findings are included in the manuscript or its supplementary files.

## Declarations

### Post-experimental animal care

After completion of the experiments, all animals were transferred to designated animal care facilities for standardized nutritional management. Health monitoring included regular observation of dietary intake and activity levels.

## References

[1] Huang GT, Gronthos S, Shi S. Mesenchymal stem cells derived from dental tissues vs. those from other sources: their biology and role in regenerative medicine. J Dent Res. 2009;88:792–806. 19767575 10.1177/0022034509340867 PMC2830488

[2] Zhang G, Zhen C, Yang J, Wang J, Wang S, Fang Y, et al. Recent advances of nanoparticles on bone tissue engineering and bone cells. Nanoscale Adv. 2024;6:1957–73. 38633036 10.1039/d3na00851g PMC11019495

[3] Zhang JY, Zhong YH, Chen LM, Zhuo XL, Zhao LJ, Wang YT. Recent advance of small-molecule drugs for clinical treatment of osteoporosis: a review. Eur J Med Chem. 2023;259:115654. 37467618 10.1016/j.ejmech.2023.115654

[4] Zhu SY, Wang PL, Liao CS, Yang YQ, Yuan CY, Wang S, et al. Transgenic expression of ephrinB2 in periodontal ligament stem cells (PDLSCs) modulates osteogenic differentiation via signaling crosstalk between ephrinB2 and EphB4 in PDLSCs and between PDLSCs and pre-osteoblasts within co-culture. J Periodontal Res. 2017;52:562–73. 27763659 10.1111/jre.12424

[5] Wang P, Wang W, Geng T, Liu Y, Zhu S, Liu Z, et al. EphrinB2 regulates osteogenic differentiation of periodontal ligament stem cells and alveolar bone defect regeneration in beagles. J Tissue Eng. 2019;10:1543343527. 10.1177/2041731419894361 PMC6918499 31897285

[6] Sun W, Ye B, Chen S, Zeng L, Lu H, Wan Y, et al. Neuro-bone tissue engineering: emerging mechanisms, potential strategies, and current challenges. Bone Res. 2023;11:65. 38123549 10.1038/s41413-023-00302-8 PMC10733346

[7] Maria OM, Heram A, Tran SD. Bioengineering from the laboratory to clinical translation in oral and maxillofacial reconstruction. Saudi Dent J. 2024;36:955–62. 39035556 10.1016/j.sdentj.2024.05.004 PMC11255950

[8] Salari SH, Saffarpour A, Jamshidi S, Ashouri M, Nassiri SM, Dehghan MM, et al. In vitro investigation of canine periodontal ligament-derived mesenchymal stem cells: a possibility of promising tool for periodontal regeneration. J Oral Biol Craniofac Res. 2023;13:403–11. 37113531 10.1016/j.jobcr.2023.03.010 PMC10127137

[9] Kim HS, Kim KH, Kim SH, Kim YS, Koo KT, Kim TI, et al. Immunomodulatory effect of canine periodontal ligament stem cells on allogenic and xenogenic peripheral blood mononuclear cells. J Periodont Implant Sci. 2010;40:265–70. 10.5051/jpis.2010.40.6.265 PMC3021166 21246016

[10] Zhu S, Liu Z, Yuan C, Lin Y, Yang Y, Wang H, et al. Bidirectional ephrinB2-EphB4 signaling regulates the osteogenic differentiation of canine periodontal ligament stem cells. Int J Mol Med. 2020;45:897–909. 31985015 10.3892/ijmm.2020.4473 PMC7015143

[11] Zayed M, Iohara K. Age related senescence, apoptosis, and inflammation profiles in periodontal ligament cells from canine teeth. Curr Mol Med. 2023;23:808–14. 35619322 10.2174/1566524022666220520124630

[12] Kania A, Klein R. Mechanisms of ephrin-Eph signalling in development, physiology and disease. Nat Rev Mol Cell Biol. 2016;17:240–56. 26790531 10.1038/nrm.2015.16

[13] Anitua E, Sanchez M, Orive G. Potential of endogenous regenerative technology for in situ regenerative medicine. Adv Drug Deliv Rev. 2010;62:741–52. 20102730 10.1016/j.addr.2010.01.001

[14] Hou J, Chen Y, Meng X, Shi C, Li C, Chen Y, et al. Compressive force regulates ephrinB2 and EphB4 in osteoblasts and osteoclasts contributing to alveolar bone resorption during experimental tooth movement. Korean J Orthod. 2014;44:320–9. 25473648 10.4041/kjod.2014.44.6.320 PMC4250666

[15] Yang Y, Ding H, Han A, Bai X, Bijle MN, Matinlinna JP, et al. *Porphyromonas gingivalis* can degrade dental zirconia. Dent Mater. 2023;39:1105–12. 37839996 10.1016/j.dental.2023.10.004

[16] Diercke K, Kohl A, Lux CJ, Erber R. Strain-dependent up-regulation of Ephrin-B2 protein in periodontal ligament fibroblasts contributes to osteogenesis during tooth movement. J Biol Chem. 2011;286:37651–64. 21880727 10.1074/jbc.M110.166900 PMC3199509

[17] Seciu AM, Craciunescu O, Stanciuc AM, Zarnescu O. Tailored biomaterials for therapeutic strategies applied in periodontal tissue engineering. Stem Cells Dev. 2019;28:963–73. 31020906 10.1089/scd.2019.0016

[18] Koch F, Meyer N, Valdec S, Jung RE, Mathes SH. Development and application of a 3D periodontal in vitro model for the evaluation of fibrillar biomaterials. BMC Oral Health. 2020;20:148. 32429904 10.1186/s12903-020-01124-4 PMC7238548

[19] Xu XY, Li X, Wang J, He XT, Sun HH, Chen FM. Concise review: Periodontal tissue regeneration using stem cells: strategies and translational considerations. Stem Cell Transl Med. 2019;8:392–403. 10.1002/sctm.18-0181 PMC6431686 30585445

[20] Chen H, Yang H, Weir MD, Schneider A, Ren K, Homayounfar N, et al. An antibacterial and injectable calcium phosphate scaffold delivering human periodontal ligament stem cells for bone tissue engineering. Rsc Adv. 2020;10:40157–70. 35520873 10.1039/d0ra06873j PMC9057516

[21] Sun Y, Zhao Z, Qiao Q, Li S, Yu W, Guan X, et al. Injectable periodontal ligament stem cell-metformin-calcium phosphate scaffold for bone regeneration and vascularization in rats. Dent Mater. 2023;39:872–85. 37574338 10.1016/j.dental.2023.07.008

[22] Lee SH, Looi CY, Chong PP, Foo JB, Looi QH, Ng CX, et al. Comparison of isolation, expansion and cryopreservation techniques to produce stem cells from human exfoliated deciduous teeth (SHED) with better regenerative potential. Curr Stem Cell Res Ther. 2021;16:551–62. 32988356 10.2174/1574888X15666200928110923

[23] Longoni A, Utomo L, van Hooijdonk IE, Bittermann GK, Vetter VC, Kruijt SE, et al. The chondrogenic differentiation potential of dental pulp stem cells. Eur Cells Mater. 2020;39:121–35. 10.22203/eCM.v039a08 32083715

[24] Omi M, Mishina Y. Roles of osteoclasts in alveolar bone remodeling. Genesis. 2022;60:e23490. 35757898 10.1002/dvg.23490 PMC9786271

[25] Qu F, Song Y, Wu Y, Huang Y, Zhong Q, Zhang Y, et al. The protective role of Ephrin-B2/EphB4 signaling in osteogenic differentiation under inflammatory environment. Exp Cell Res. 2021;400:112505. 33516666 10.1016/j.yexcr.2021.112505

[26] Zhao C, Irie N, Takada Y, Shimoda K, Miyamoto T, Nishiwaki T, et al. Bidirectional ephrinB2-EphB4 signaling controls bone homeostasis. Cell Metab. 2006;4:111–21. 16890539 10.1016/j.cmet.2006.05.012

[27] You C, Zhao K, Dammann P, Keyvani K, Kreitschmann-Andermahr I, Sure U, et al. EphB4 forward signalling mediates angiogenesis caused by CCM3/PDCD10-ablation. J Cell Mol Med. 2017;21:1848–58. 28371279 10.1111/jcmm.13105 PMC5571521

[28] Barros D, Conde-Sousa E, Goncalves AM, Han WM, Garcia AJ, Amaral IF, et al. Engineering hydrogels with affinity-bound laminin as 3D neural stem cell culture systems. Biomater Sci. 2019;7:5338–49. 31620727 10.1039/c9bm00348g PMC6864240

[29] Wang W, Yuan C, Geng T, Liu Y, Zhu S, Zhang C, et al. EphrinB2 overexpression enhances osteogenic differentiation of dental pulp stem cells partially through ephrinB2-mediated reverse signaling. Stem Cell Res Ther. 2020;11:40. 31996240 10.1186/s13287-019-1540-2 PMC6990579

[30] Heng BC, Wang S, Gong T, Xu J, Yuan C, Zhang C. EphrinB2 signaling enhances osteogenic/odontogenic differentiation of human dental pulp stem cells. Arch Oral Biol. 2018;87:62–71. 29272761 10.1016/j.archoralbio.2017.12.014

[31] Wang W, Yuan C, Geng T, Liu Y, Zhu S, Zhang C, et al. Lipopolysaccharide inhibits osteogenic differentiation of periodontal ligament stem cells partially through toll-like receptor 4-mediated ephrinB2 downregulation. Clin Oral Invest. 2020;24:3407–16. 10.1007/s00784-020-03211-w 31974644

[32] Yuan C, Wang P, Zhu S, Zou T, Wang S, Xu J, et al. EphrinB2 stabilizes vascularlike structures generated by endothelial cells and stem cells from apical papilla. J Endod. 2016;42:1362–70. 27451120 10.1016/j.joen.2016.05.012

