# Supplementary figures and images for: Transgenic high expression of EphB4 in canine periodontal ligament stem cells modulates osteogenic differentiation and migration in cPDLSCs
Source: Biomed Eng Online. 2026 Feb 9;25:63. doi: 10.1186/s12938-026-01533-6 (PMC13123108; doi:10.1186/s12938-026-01533-6)

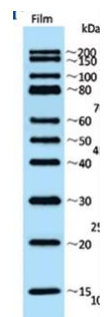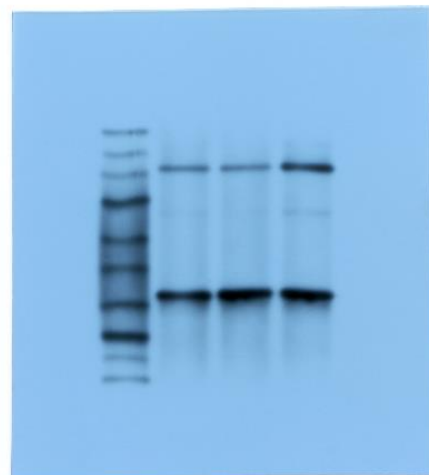

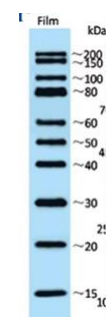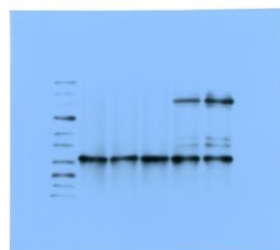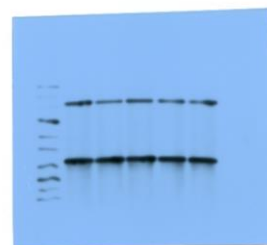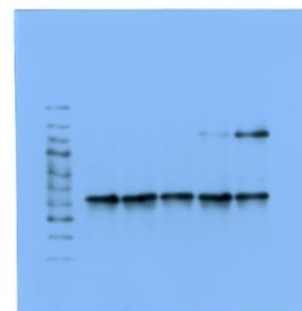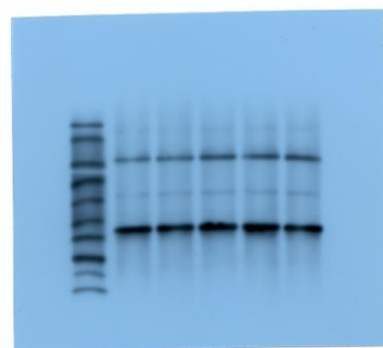

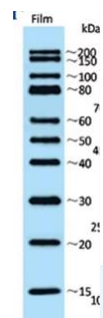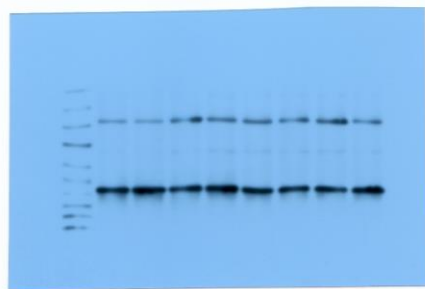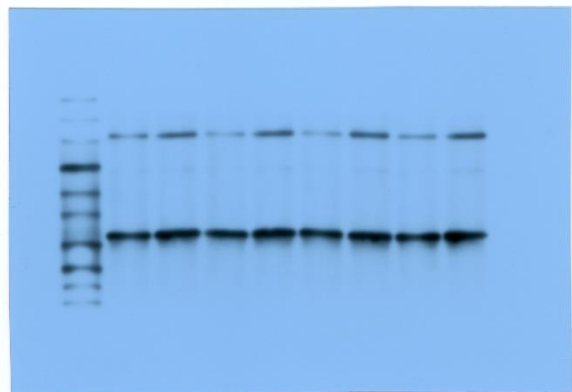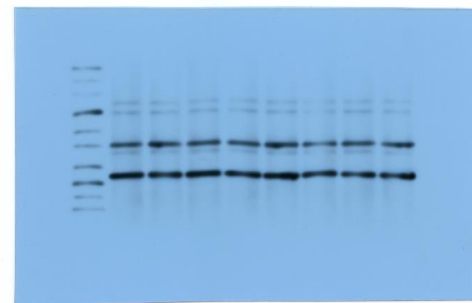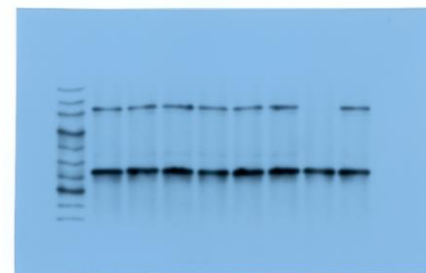

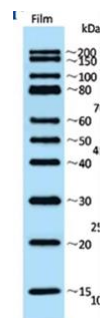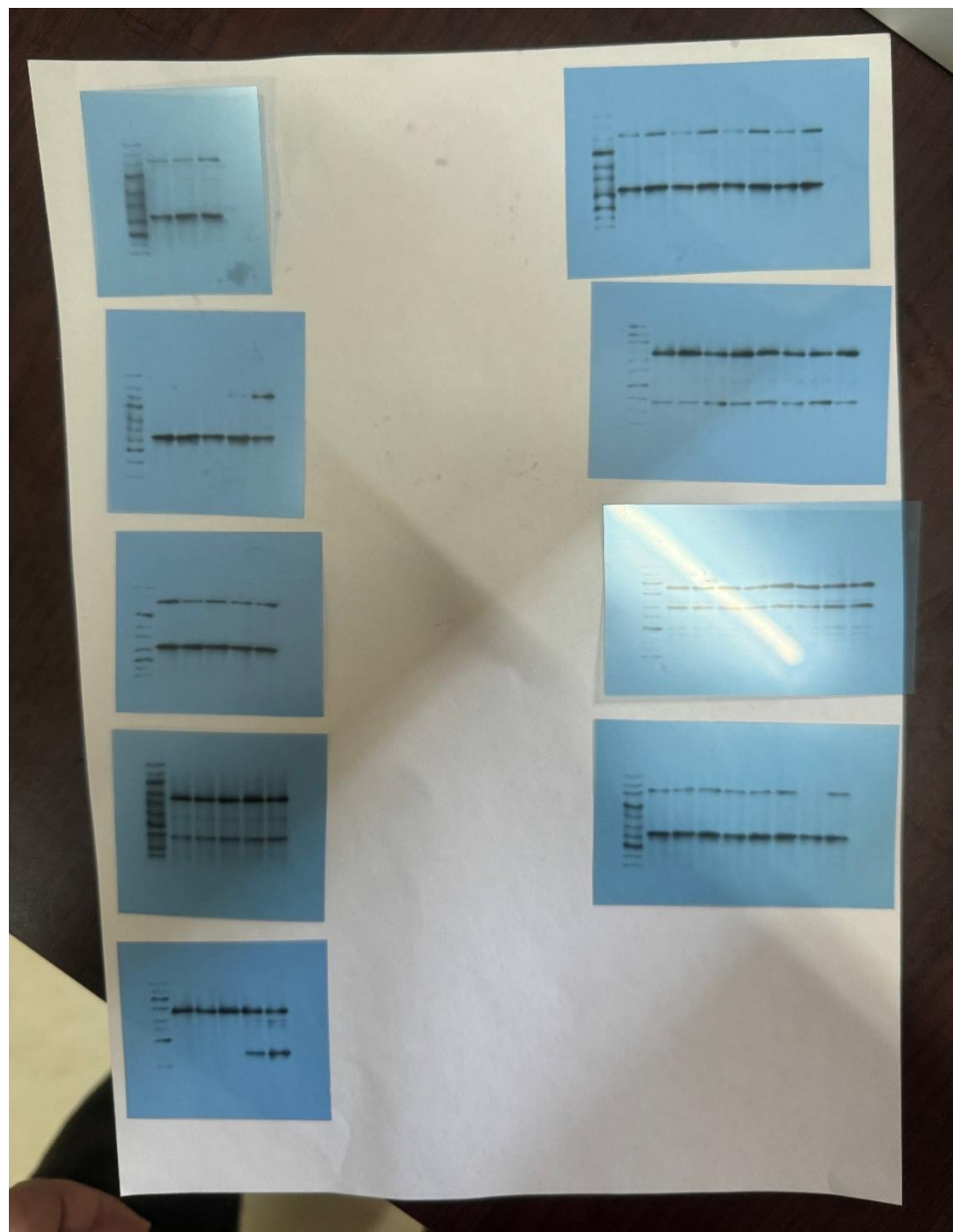

Supplement: Supplementary file 3 — Supplementary Material 3. [file 12938_2026_1533_MOESM3_ESM.pdf]
